# Establishment and comparison of RF-RAA and qPCR for rapid detection of Chinese soft-shelled turtle adenovirus (CSTAdV)

**DOI:** 10.1186/s12985-025-02895-4

**Published:** 2025-08-18

**Authors:** Feiyan Tian, Zhigao Zhan, Jianming Pei, Haili Huang, Zemao Gu

**Affiliations:** 1 Department of Aquatic Animal Medicine, College of Fisheries, Huazhong Agricultural University, Wuhan, 430070 China; 2 Jiangxi Provincial Center for Agricultural Technical Extension, Nanchang, 330046 China; 3 Institute of Microbiology, Jiangxi Academy of Sciences, Nanchang, 330029 China

**Keywords:** Chinese soft, Shelled turtle adenovirus, Recombinase, Aided amplification, Real, Time quantitative polymerase chain reaction, Rapid detection

## Abstract

**Background:**

Chinese soft-shelled turtle adenovirus (CSTAdV) is a new virus discovered recently that infects farmed Chinese soft-shelled turtle. In order to investigate its epizootiology and meet the requirements of timely prevention and control, it is imperative to establish an efficient diagnostic assay for CSTAdV.

**Results:**

In this study, on-site diagnostic real-time fluorescence Recombinase-aided amplification (RF-RAA) and real-time quantitative polymerase chain reaction (qPCR) detection methods were established based on the specific sequence of the viral DNA polymerase gene. The results showed that the sensitivity of the CSTAdV RF-RAA assay and qPCR assay was 1.0 × 10^2^ copies/μL within 20 min at 42 °C, and 1.0 × 10^1^ copies/μL in approximately 60 min for detecting plasmid pUC57-CSTAdV, respectively. Both the RF-RAA assay and the qPCR assay were highly specific for CSTAdV, with no cross-reaction with Soft-shelled turtle iridovirus, *Trionyx sinensis* hemorrhagic syndrome virus, *Citrobacter freundii*, *Aeromonas veronii, Aeromonas hydrophila, Morganella morganii*, and *Vibrio cholerae*. A total of 107 clinical specimens of Chinese soft-shelled turtle were tested by the RF-RAA and qPCR assays. The qPCR results were consistent with adenoviral consensus nested PCR published previously, whereas the RF-RAA assay exhibited diagnostic sensitivity (DSe) and diagnostic specificity (DSp) of 95.45% and 100%, respectively. The findings suggest that the newly developed RF-RAA and qPCR assays exhibit high accuracy in detecting CSTAdV in clinical specimens.

**Conclusions:**

Therefore, the RF-RAA and qPCR assays provide two novel alternatives for simple, sensitive and specific identification of CSTAdV for pathogen screening in the field and quantitative analysis in the laboratory.

## Introduction

Adenoviridae is a family of viruses with non-enveloped, icosahedral virions containing linear dsDNA genomes of 25–48 kb. Members of this family infect a wide range of vertebrate hosts, from fish to humans, and are categorized into six genera: *Aviadenovirus*, *Barthadenovirus (*formerly *Atadenovirus)*, *Ichtadenovirus*, *Mastadenovirus*, *Siadenovirus*, and *Testadenovirus* [1, 2]. Among them, strains of genus *Testadenovirus* occur in turtles, *Ichtadenovirus* includes a single species infecting fish, and *Siadenovirus* infects birds, frogs and tortoises [2–4]. The severity of adenovirus infections ranges from subclinical to lethal [1].

In 2023, an emerging disease syndrome in farmed Chinese soft-shelled turtle (*Pelodiscus sinensis*) in Jiangxi Province, China, was characterized by reduced egg production, slight epithelial hemorrhage on the plastron, and severely hemorrhagic intestine with undigested beige residual. Preliminary evidence suggested the association of a novel adenovirus (provisionally designated Chinese soft-shelled turtle adenovirus 1, CSTAdV-1) with the disease. Genomic analysis by Tian et al. revealed that CSTAdV-1 has a 25,548 bp double-stranded DNA genome and was suggested to be classified into the *Siadenovirus* genus [5]. Turtles are predominantly linked to testadenoviruses, although Barthadenovirus and siadenoviruses have also been identified in these species [6–8]. Among these, the sulawesi tortoise adenovirus, which appears to be associated with *Siadenovirus* based on phylogenetic analyses of partial DNA polymerase gene sequences, caused severe systemic disease with very high mortality rate [8].

From the perspective of preventing major aquaculture losses, it is imperative to establish diagnostic methods for this new virus as soon as possible to effectively investigate its epizootiology and meet the requirements of timely prevention and control. Rapid on-site diagnosis is conducive to the timely detection and control of animal diseases. Recombinase-aided amplification (RAA) is a novel isothermal amplification technology that has garnered significant attention due to its efficient and specific DNA amplification capabilities under isothermal conditions of 37 °C-42 °C within 30 min [9]. RAA provides different kinds of end-point detection formats, such as real-time fluorescence, lateral flow dipstick, and agarose gel electrophoresis. In recent years, the RAA technique has emerged as a promising on-site tool in molecular biology for rapid pathogen diagnosis utilizing simple equipment, such as H5 subtype avian influenza virus [10] and duck circovirus [11]. Quantitative detection of pathogens in infected animals is one of the most important means for assessing disease progression. Real-time quantitative polymerase chain reaction (qPCR) detects the amount of gene amplification by capturing fluorescence signals, and has high detection sensitivity, specificity and fast detection speed [12]. it is the most prevalent technique for detecting and quantifying pathogens of aquatic animals in 2023 edition of the *Manual of Diagnostic Tests for Aquatic Animals* (World Organisation for Animal Health, WOAH). In this study, we aimed to develop two highly sensitive and specific assays: a real-time fluorescence RAA (RF-RAA) assay for on-site rapid detection and a qPCR assay for the precise quantification of CSTAdV. Targeting the DNA polymerase gene of CSTAdV, we designed primers and probes for RF-RAA and qPCR assays to detect CSTAdV. We optimized reaction conditions, evaluated sensitivity and specificity, and validated utility through clinical sample testing.

## Materials and methods

### Pathogens and clinical specimens

CSTAdV-1 [5], *Citrobacter freundii* (Cf) LCJY-002 [13], *Aeromonas veronii* (Av) Av-202201, *Aeromonas hydrophila* (Ah) Ah-202401 and *Morganella morganii* (Mm) Mm-202407 were isolated and preserved in our laboratory. Soft-shelled turtle iridovirus (STIV), *Trionyx sinensis* hemorrhagic syndrome virus (TSHSV) were generously provided by Dr. Liu Hong of Shenzhen customs district, P.R. China, and Dr. Liu Li of Institute of hydrobiology, Zhejiang Academy of Agricultural sciences, P.R. China, respectively. *Vibrio cholerae* (*Vc*) was provided by the Nanchang City Center for Disease Control and Prevention, jiangxi Province, China. During April to June in 2024, a total of 107 clinical internal organ tissues (liver, kidney, spleen) from diseased Chinese soft-shelled turtles were collected from farms in Nanfeng County of Fuzhou city, Jiangxi Province, China. This county produces more than a half of the Chinese soft-shelled turtle eggs and seedlings in China [14]. The clinical features of these diseased Chinese soft-shelled turtles included: putrid Skin, hepatomegalia, intestinal bleeding, intestinal obstruction, parotiditis etc.

### DNA/RNA Extraction and Generation of DNA Standard

Total DNA/RNA of all viruses was extracted from 100 μL of virus suspensions using the TaKaRa MiniBEST Viral RNA/DNA Extraction Kit (Beijing, China) according to the manufacturer’s instructions. The cDNA of TSHSV was synthesized using the TaKaRa PrimeScript™ 1 st strand cDNA Synthesis Kit (Beijing, China). Bacterial genomic DNA was extracted using the TaKaRa MiniBEST Bacteria Genomic DNA Extraction Kit (Beijing, China) according to the manufacturer's instructions. Total DNA of 107 clinical internal organ tissues (liver, kidney, spleen) from diseased Chinese soft-shelled turtles was extracted from 200 μL of tissue suspensions using the Qiagen DNeasy Blood & Tissue Kit (Hilden, NRW, Germany) according to the manufacturer’s standardized instructions, the concentration and quality of DNA were tested using BioPhotometer Plus Nucleic acid/Protein analyzer (Eppendorf, Germany) after DNA extraction. The CSTAdV-1 DNA polymerase gene segments (3,324 bp, GenBank accession no. PQ083072) were synthesized by Sangon Biotech (Shanghai, China) and cloned into a pUC57 vector, designated as pUC57-CSTAdV. The pUC57-CSTAdV standard plasmid DNA was extracted using TIANGEN TIANprep Mini Plasmid Kit (Beijing, China) and then measured by BioPhotometer Plus Nucleic acid/Protein Analyzer (Eppendorf, Germany), The DNA copy number was calculated using the following equation: DNA copy number = (M × 6.02 × 10^23^ × 10^–9^)/(n × 660). The calculation result is 1.0 × 10^9^ copies/μL. The pUC57-CSTAdV standard plasmid DNA was subsequently diluted tenfold with TE buffer. Both DNA samples were stored at −80 °C until use.

### Design of the Primer and Probe of RF-RAA and qPCR

Based on the complete genome sequence of CSTAdV-1 (GenBank accession no. PQ083072), the DNA polymerase gene was selected for primer and probe design after comparative analysis of conserved regions across previously identified positive samples. In accordance with the guidelines provided by ZC Bioscience RAA kits (Hangzhou Zhongce Bio-Sci & Tech Co. Ltd., China), three forward primers, two reverse primers, and two probes were designed to identify the most efficient combination for RF-RAA assays. Additionally, forward and reverse primers and along with a probe were designed for qPCR analysis using Primer Premier 5.0 software. All designed primers and probes were subjected to BLAST analysis to verify their specificity. Subsequently, they were synthesized by Sangon Biotech (Shanghai, China). The sequences of the designed primers and probes were summarized in Table 1.

**Table 1:** Primers and probes for CSTAdV Real-Time qPCR and RF-RAA

Method	**Primer name**	**sequences (5'−3')**
CSTAdV qPCR	CSTAdV-qPCR -F	ATGATTTGAGAAACCGTCAG
	CSTAdV- qPCR -R	TGATTTTGGAAAGCATAGAC
	CSTAdV- qPCR -P	FAM-CACATTTAGATTCGCATTCTACGGCC-TAMRA
CSTAdV RF-RAA	CSTAdV-RAA-F1	GAGATAATCATTGGTTTAATTTCGTGGTCG
	CSTAdV-RAA-F2	AATGGAGATAATCATTGGTTTAATTTCGTG
	CSTAdV-RAA-F3	TTGATTGTAATAGTTCTATGGCCTGTTCTT
	CSTAdV-RAA-R1	TGTTTATGACATCTGCGGTATGTATGCTAG
	CSTAdV-RAA-R2	TCTATGTTTATGACATCTGCGGTATGTATG
	CSTAdV-RAA-P1	TAATAGTTCTATGGCCTGTTCTTTTTCT[FAM-dT]T[THF]T[BHQ1-dT]GCTGATAGGAATG-P
	CSTAdV-RAA-P2	ATGCTAGTGCTTTAACACACCCAATGCCA[FAM-dT] [THF] [BHQ1-dT]GGCATTCCTATCAG-P
Adenoviral consensus nested PCR ( Wellehan et al., 2004 )	AdV-F1	TNMGNGGNGGNMGNTGYTAYCC
	AdV-F2	GTDGCRAANSHNCCRTABARNGMRTT
	AdV-R1	GTNTWYGAYATHTGYGGHATGTAYGC
	AdV-R2	CCANCCBCDRTTRTGNARNGTRA

### Optimization of reaction conditions for RF-RAA

The probe-based CSTAdV RF-RAA assay was performed using a fluorescence detection device T16-ISO (TwistDx, United Kingdom) in a 50 μL reaction volume. The reaction mixture was prepared using the ZC Bioscience RAA exo kit (Hangzhou Zhongce Bio-Sci & Tech Co. Ltd., China), which included 25 μL of A buffer (rehydration buffer), 5 μL of template DNA, 2 μL each of forward and reverse primers (10 μM, Sangon Biotech, China), 0.6 μL of RAA exo probe (10 μM, Sangon Biotech, China), 12.9 μL of ddH_2_O and 2.5 μL of B buffer (280 mM, magnesium acetate). The reaction time was carried out for 20 min. To determine the optimal reaction conditions, different combinations of primers and probes were tested to amplify 1.0 × 10^7^ copies/μL of pUC57-CSTAdV DNA as the template, with three replicates for each condition. Additionally, the assay was performed at different incubation temperatures (35 °C, 37 °C, 39 °C, 40 °C, and 42 °C). Fluorescence intensity was measured using the FAM channel (excitation 470 nm and detection 520 nm) for 20 min.

### Optimization of reaction conditions for qPCR

The TaqMan probe-based CSTAdV qPCR assay was performed on a Gentier 96E quantitative fluorescence instrument (Tianlong, China) in a 20 μL reaction volume using the TaKaRa Probe qPCR Mix kit (Beijing, China). Using 1.0 × 10^7^ copies/μL of pUC57-CSTAdV DNA as the template, the annealing temperature was optimized by testing a gradient range of 50–62 °C. Additionally, different final concentrations of primers (0.1–0.8 μmol/L) and probe (0.1–0.5 μmol/L) were tested to determine the optimal combination that resulted in the lowest critical cycle number (Ct) and the highest relative fluorescence units (RFU).

### Specificity of the RF-RAA and qPCR assays

The specificity of the developed CSTAdV RF-RAA and qPCR assays were evaluated by detecting a panel of pathogens, including CSTAdV-1, STIV, TSHSV, Cf, Av, Ah, Mm, and Vc. All detection assays were performed in triplicate, using pUC57- CSTAdV (1.0 × 10^7^ copies/μL) as the positive control and ultrapure water as the negative control.

### Sensitivity of the RF-RAA and qPCR assays

To evaluate the sensitivity of RF-RAA and qPCR assays, tenfold serial dilutions of the standard plasmid pUC57-CSTAdV DNA (ranging from 1.0 × 10^6^ copies/μL to 1.0 copy/μL) were used as templates to determine the detection limits. All detection assays were performed in quintuplicate, with ultrapure water serving as the negative control. Standard curves for the qPCR assay were generated based on the concentration gradient of the diluted plasmid and Ct values.

### Repeatability of the RF-RAA and qPCR assays

The repeatability of the CSTAdV RF-RAA and qPCR assays was evaluated using pUC57-CSTAdV plasmid standards at concentrations of 1.0 × 10^6^ copies/μL and 1.0 × 10^3^ copies/μL as templates. Intra-group repeatability tests were performed with three replicates for each concentration. Additionally, three independent inter-group repeatability tests were conducted under identical conditions at 7-day intervals to further assess the repeatability of the methods.

### Practical application of the clinical samples

The practicality of both the CSTAdV RF-RAA and qPCR assays was evaluated using 107 archived DNA samples extracted from clinical internal organ tissues of diseased turtles. The results were compared with those obtained from the adenoviral consensus nested PCR assay, with both the reaction system and amplification protocol of the nested PCR being strictly implemented according to the referenced methodology [15]. The positive control was pUC57-CSTAdV plasmid DNA (1.0 × 10^7^ copies/μL), the negative control was DNA from healthy Chinese soft-shelled turtles, and the blank controls was ultrapure water. Each assay was performed in duplicate. Diagnostic sensitivity (DSe) and diagnostic specificity (DSp) were calculated following the guidelines provided in the 2023 edition of the Manual of Diagnostic Tests for Aquatic Animals (WOAH).

## Results

### Optimization results of CSTAdV RF-RAA and qPCR assays

**Fig. 1:**
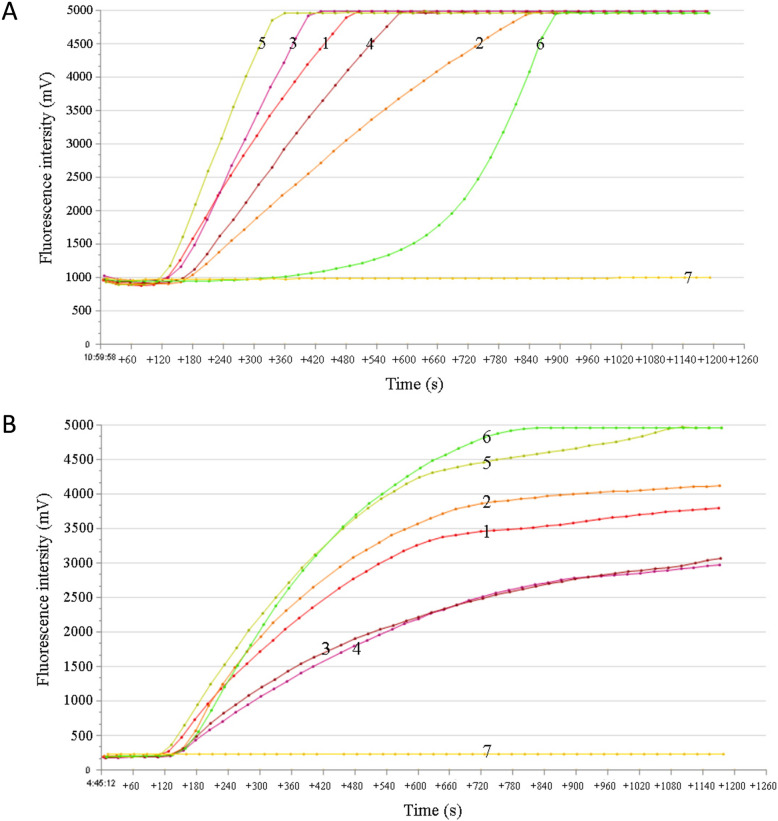
Screening primers and probe for real-time fluorescence Recombinase-aided amplification (RF-RAA) detection of Chinese soft-shelled turtle adenovirus (CSTAdV). **A** Amplification curves of the combinations of probe CSTAdV-RAA-P1 with forward/reverse primers. **B** Amplification curves of the combinations of probe CSTAdV-RAA-P2 with forward/reverse primers. 1: F1-R1; 2: F1-R2; 3: F2-R1; 4: F2-R2; 5: F3-R1; 6: F3-R2; 7: Negative control

The designed RF-RAA primers and probes were arranged and combined to perform the CSTAdV RF-RAA assay. The relative performances of the candidate primer and probe groups were evaluated and compared. Combinations involving the probe CSTAdV-RAA-P1 with forward/reverse primers were discontinued due to excessive fluorescence intensity (≥ 1,000 mV) observed during initial amplification (Fig. 1A). Subsequently, an additional probe, CSTAdV-RAA-P2, was designed to target the complementary strand of the DNA polymerase sequence. The results demonstrated that the combinations of probe CSTAdV-RAA-P2 with primers CSTAdV-RAA-F3/R2 yielded the highest amplification efficiency on the fluorescence detection device T16-ISO (Fig. 1B). The optimal temperature was found to be 42 °C (data not shown). Therefore, the combination of primers and probe CSTAdV-RAA-F3/R2/P2 were selected for the CSTAdV RF-RAA assay at the optimized temperature of 42 °C for further validation.

For qPCR optimization, reactions were performed in a 20 μL reaction system containing 10 μL of 2 × probe qPCR mix, 600 nM of each primer (CSTAdV-qPCR-F/R), 200 nM TaqMan probe (CSTAdV-qPCR-P), and 2 μL DNA template using the Probe qPCR Mix kit (TaKaRa, Beijing, China). The thermal cycling protocol consisted of an initial denaturation step at 95 °C for 1 min, followed by 40 cycles of amplification with denaturation at 95 °C for 15 s and annealing at 55 °C for 60 s (data not shown).

### Specificity analysis

**Fig. 2:**
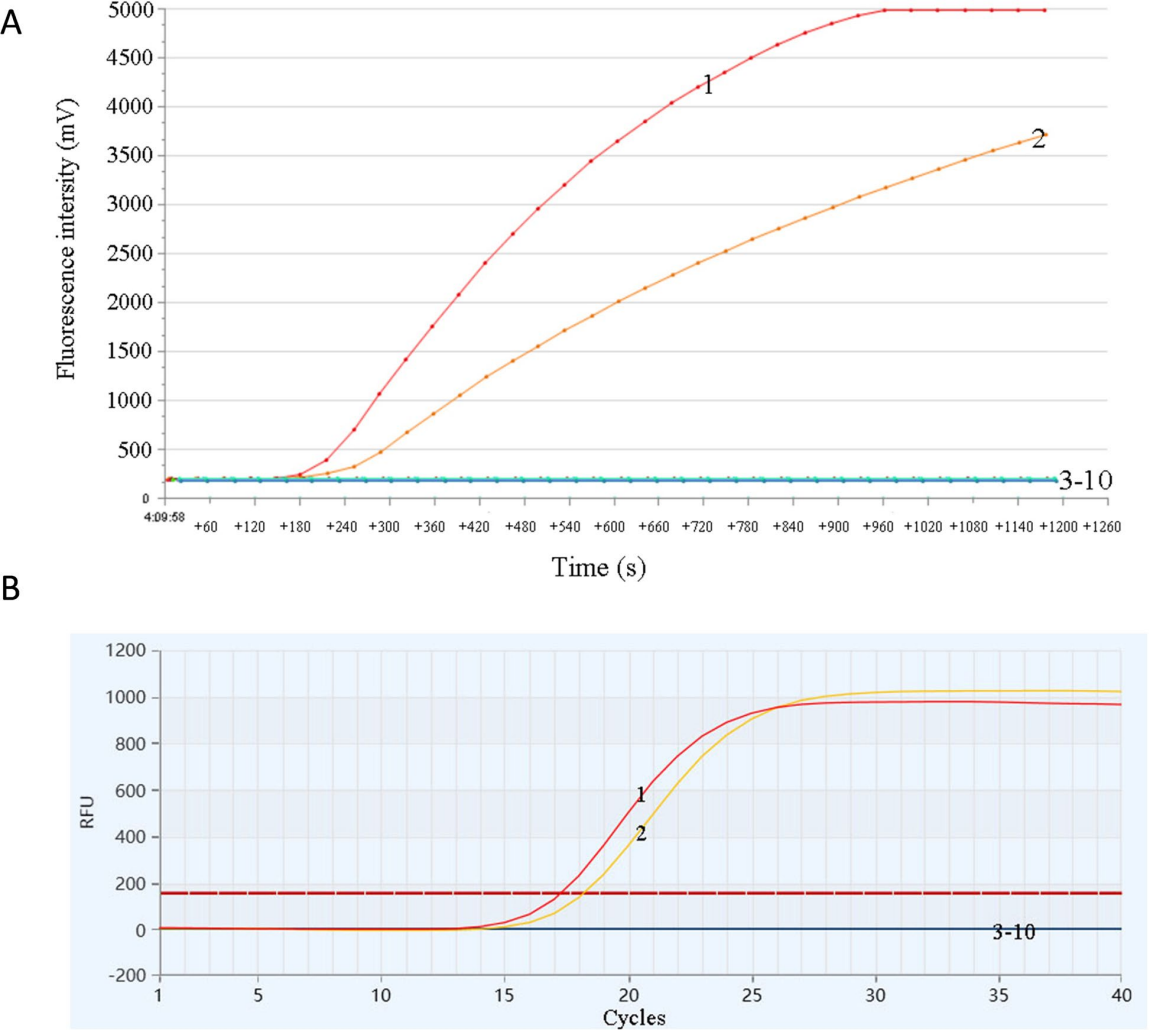
Specificity test results of the CSTAdV RF-RAA assay and the CSTAdV qPCR assay using total DNA extracted from pUC57-CSTAdV, CSTAdV-1 and other pathogens. **A** the CSTAdV RF-RAA assay; **B** the CSTAdV qPCR assay. 1: pUC57- CSTAdV; 2: CSTAdV-1 genomic DNA; 3: STIV; 4: TSHSV; 5: Cf; 6: Av; 7: Ah; 8: Mm; 9: Vc; 10: Negative control

No cross-reactivity was observed with STIV*,* TSHSV, Cf, Av, Ah, Mm, Vc, or the negative control. Positive signals were only detected with CSTAdV-1 genomic DNA and pUC57-CSTAdV (Fig. 2A and B). These results indicate that the established CSTAdV RF-RAA and qPCR detection methods exhibit excellent specificity.

### Sensitivity analysis

**Fig. 3:**
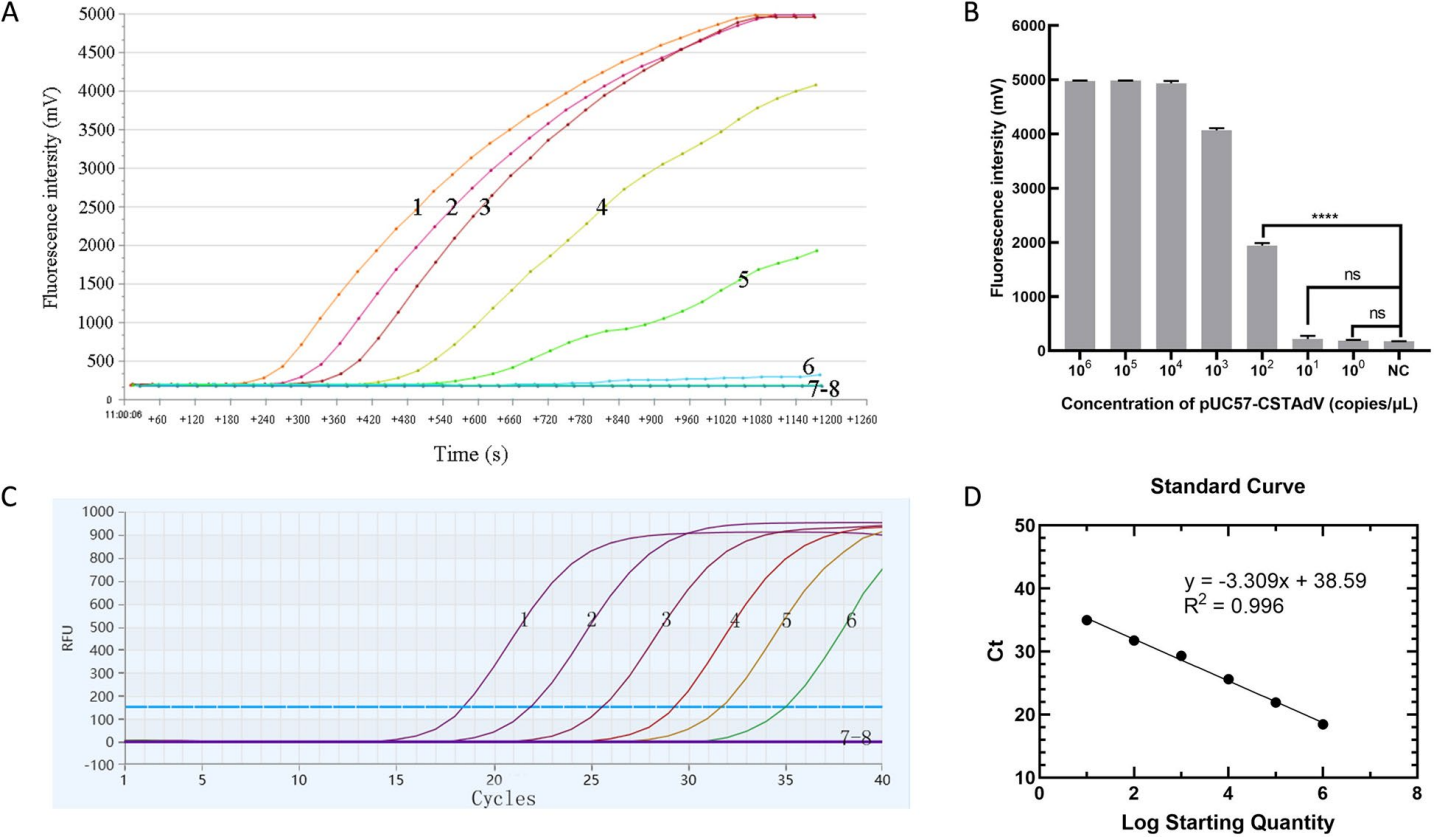
Evaluation of the sensitivity of the CSTAdV RF-RAA assay and the CSTAdV qPCR assay for detecting pUC57- CSTAdV DNA. **A** the CSTAdV RF-RAA assay. **B** Statistical Significance Analysis of CSTAdV RF-RAA assay. NC: Negative control; ****: *P* < 0.0001; ns: No significant; **C** the CSTAdV qPCR assay. **D** The standard curve of qPCR. 1–7: a dilution range from 1.0 × 10^6^–1.0 copies/μL of pUC57-CSTAdV DNA; 8: Negative control

The sensitivities of the newly established RF-RAA and qPCR assays were evaluated using recombinant plasmid dilutions from 1.0 × 10^6^ copies/μL to 1.0 copy/μL. As shown in Fig. 3A and C, the real-time fluorescence signals exhibited a robust response to target DNA concentrations ranging from 1.0 × 10^6^ copies/μL to 1.0 × 10^2^ copies/μL. However, weaker signals were observed at the target DNA concentration of 1.0 × 10^1^ copies/μL for RF-RAA, while the sensitivity of the CSTAdV qPCR assay was 1.0 × 10^1^ copies/μL for detecting plasmid pUC57-CSTAdV. Overall, both methods demonstrated high sensitivity, enabling effective amplification even at low concentrations.

A comparison of the amplification curves between RF-RAA and qPCR (Fig. 3A and C) revealed that the qPCR amplification curve consistently shifted backward with decreasing target concentration, whereas the RF-RAA amplification curve did not exhibit exhibit a similar proportional shift. Additionally, the amplification signal was weak and less reliable at the low concentration of 1.0 × 10^1^ copies/μL (Fig. 3B). These findings suggest that the sensitivity of RF-RAA for detecting plasmid pUC57-CSTAdV DNA is slightly lower than that of qPCR. The qPCR standard curve demonstrated a strong linear correlation between plasmid concentration and the Ct value (R^2^ = 0.996) (Fig. 3D), confirming the reliability of the developed qPCR assay for accurately quantifying target DNA using a standard curve. In terms of reaction speed, RF-RAA provided amplification results within 20 min, while qPCR required approximately 60 min to complete. Thus, the overall amplification rate of RF-RAA was faster than that of qPCR.

### Repeatability analysis

**Fig. 4:**
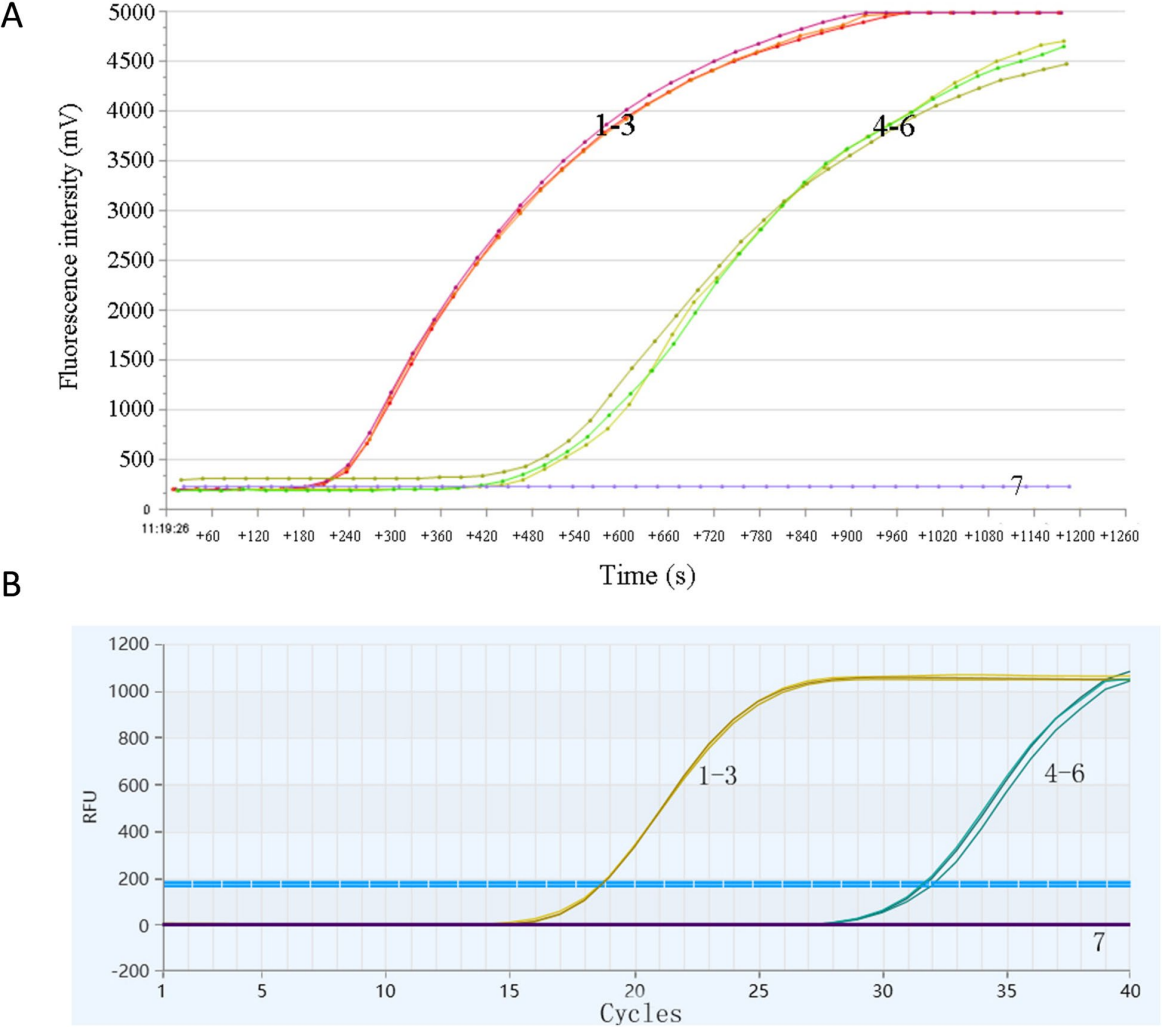
Repeatability of the CSTAdV RF-RAA assay (**A**) and the CSTAdV qPCR (**B**) assay for detecting pUC57-CSTAdV DNA. 1–3: dilution were 1.0 × 10^6^ copies/μL; 4–6: dilution were 1.0 × 10^3^ copies/μL; 7: Blank control

The CSTAdV RF-RAA and qPCR assays developed in this study were employed to assess intra- and inter-group repeatability. Standard plasmids with concentration of 1.0 × 10^6^ copies/μL and 1.0 × 10^3^ copies/μL were used as templates. The results indicated that the amplification curves for templates of the same concentration were consistent across both intra-group and inter-group tests, with only minor variations observed (Fig. 4A and B). These findings demonstrate that the CSTAdV RF-RAA and qPCR assays exhibit excellent reproducibility and high reliability.

### Detection of CSTAdV in clinical samples using RF-RAA and qPCR

Among the 107 DNA samples, 44 tested positive and 63 tested negative using the adenoviral consensus nested PCR assay [15], and all positive samples were confirmed by sequencing. The results of the qPCR assay were consistent with those of the adenoviral consensus nested PCR assay (Table 2). Consequently, the DSe and DSp of the qPCR assay for the clinical samples were both 100% compared to the nested PCR assay. The RF-RAA assay successfully detected 42 CSTAdV-positive samples, with only 2 cases misdiagnosed as negative, the DSe and DSp of the RF-RAA assay were 95.45% and 100%, respectively. These results demonstrate that both the RF-RAA and qPCR assays exhibit high accuracy in detecting CSTAdV in clinical samples.

**Table 2:** Comparative evaluation of RF-RAA, qPCR, and nested PCR for DNA detection in diseased Chinese soft-shelled turtles

Methods	NO. of positive samples	NO. of negative samples	positive rate
RF-RAA	42	65	39.25%
qPCR	44	63	41.12%
Nested PCR	44	63	41.12%

## Discussion

The Chinese soft-shelled turtle, *Pelodiscus sinensis* or *Trionyx sinensis*, belongs to the class *Reptilia*, order *Testudinata*, family *Trionychidae* and genus *Pelodiscus*. It is widely distributed in China, Japan, Thailand and other Asian countries [16, 17]. Due to its delicious flavor and rich nutritional value, the production of Chinese soft-shelled turtle has been increasing over the years, and this species plays an important role in aquaculture in southern and central China. However, the cultivation of soft-shelled turtles has been significantly impacted by diseases. The presence of various pathogens, including viruses and bacteria, along with the similarity of clinical symptoms [5, 13, 18–22], makes it difficult for front-line technicians to accurately diagnose infections based solely on clinical observations. Therefore, it is crucial to develop rapid and accurate methods for detecting pathogens in diseased turtles.

CSTAdV was previously identified from clinical samples of diseased Chinese soft-shelled turtle using cell culture techniques in our earlier studies [5]. Although cell culture is widely regarded as the gold standard for detecting most viruses, it is characterized by high costs, labor-intensive procedures, and time-consuming processes. Therefore, it is not suitable for large-scale sample detection in epidemiological investigations or disease prevention and control. Nucleic acid amplification technology has become the most commonly recommended method for pathogen detection in 2023 edition of the *Manual of Diagnostic Tests for Aquatic Animals* (WOAH) due to its advantages of high precision, low cost, rapid processing, and high throughput.

In order to achieve rapid diagnosis in the field and quantitative analysis in laboratories for CSTAdV, this study aimed to establish reliable RF-RAA and qPCR detection methods for CSTAdV. Maximum likelihood analysis based on DNA polymerase amino acid sequence is one of the criteria recommended by International Committee on Taxonomy of Viruses (ICTV) for the demarcation of adenovirus genera/species [1]. To improve the specificity of the CSTAdV detection methods, the DNA polymerase gene was selected for primer and probe design. In the RF-RAA experiments, we designed three upstream primers, two downstream primers, and two probes to screen for the best combination of primers and probe, due to the lack of established principles and experience in designing of primers and probes for the RF-RAA method. The probe CSTAdV-RAA-P2, combined with primer pair CSTAdV-RAA-F3/R2, demonstrated the highest amplification efficiency and the lowest fluorescence intensity (≤ 200 mV) during initial amplification phase, making it suitable for RF-RAA systems. By comparing and analyzing the structural characteristics of probe CSTAdV-RAA-P1and probe CSTAdV-RAA-P2 (Table 1) and the amplification results of their combinations with upstream and downstream primers (Fig. 1A and B), we summarized the following experience for RF-RAA probe design: the closer and more symmetrical the distance between the flanking dT-fluorophore and the corresponding dT-quencher group, the lower the fluorescence intensity value during initial amplification. The RF-RAA reaction can be conducted within a broad temperature range of 37–42 °C, with the amplification curve exhibiting its peak fluorescence value at 42 °C. Therefore, the combination of primers and probe CSTAdV -RAA-F3/R2/P2 was utilized in the CSTAdV RF-RAA assay at an optimal temperature (42 °C) for further validation. The primers and probe for qPCR were designed in accordance with the design principles of primer and probe for qPCR. A fluorescence qPCR assay for the detection of CSTAdV was established by optimizing the annealing temperature and the concentration of primers and probe. A strong linear relationship was observed between the logarithm of the concentration and the Ct value, and the established standard curve of the CSTAdV qPCR assay accurately reflect the amplification of the target product.

Both the developed CSTAdV RF-RAA and qPCR assays are highly specific for detection of CSTAdV, as only the standard plasmid pUC57-CSTAdV and DNA extracted from the diseased soft-shelled turtle with CSTAdV produced positive results in the ampliffcation curve, indicating their potential possibility as novel tools for differential diagnosis. Sensitivity tests revealed that the qPCR assay could detect plasmid pUC57-CSTAdV DNA at concentrations as low as 1.0 × 10^1^ copies/μL. The qPCR standard curve showed a high correlation coefficient (R^2^ = 0.996), and the regressive equation was Ct =  − 3.309 × lg (CSTAdV DNA copies) + 38.59 (Fig. 3D). The RF-RAA assay demonstrated a statistically significant difference in fluorescence values compared to the negative control at a concentration of 1.0 × 10^2^ copies/μL (P < 0.01). However, no significant difference was observed at 1.0 × 10^1^ copies/μL. Thus, the detection limit of the RF-RAA assay was determined to be 1.0 × 10^2^ copies/μL (Fig. 3B). Additionally, the RF-RAA assay currently cannot achieve quantitative analysis. Comparative analysis of the two assays demonstrates that qPCR exhibits superior sensitivity and stability for detecting trace amounts of target DNA compared to RF-RAA. The use of qPCR for detecting trace pathogens in soft-shelled turtle is highly recommended for laboratory settings. Although RF-RAA is slightly less sensitive and stable than qPCR and cannot be quantitative analysis, it can amplify the target at a constant temperature more rapidly. The RF-RAA device is simpler, smaller and more portable than the qPCR instrument, and it can be conveniently used in the field as it can be powerd by a battery. These features made RF-RAA more suitable for on-site testing or rapid diagnosis in less-equipped or mobile laboratories. Therefore, RF-RAA is ideal for rapid detection in the field, while qPCR is better suited for quantifying samples with low target concentrations.

In this study, the newly established qPCR and RF-RAA assays did not adopt an internal control for monitoring DNA template quality. Firstly, all samples were extracted using a standardized protocol, the extracted DNA was quantified using an Eppendorf BioPhotometer Plus nucleic acid/protein analyzer. Secondly, preliminary screening was performed using the adenoviral consensus nested PCR, and all positive samples were confirmed by sequencing. Thirdly, emerging evidence indicates that adenoviruses can cross host species barriers [23]. As a newly identified adenoviral pathogen, CSTAdV may also exhibit cross-species transmission potential. Consequently, the identification of a suitable host internal control presented difficulties. Based on the above considerations and referring to the convention in the World Organization for Animal Health (WOAH) Manual that most qPCR detection methods for aquatic animal pathogens do not set internal reference controls, this study also did not set internal reference controls. However, in practical applications, internal controls are essential for reliable pathogen detection in trace-quantity samples.

Molecular epidemiological investigation of 107 diseased turtle specimens identified a high detection rate of the novel CSTAdV, indicating widespread adenovirus infections among turtles. This finding is supported by previous reports of adenoviral infections in Sulawesi tortoises [8]. However, the geographical limitations of the sampled specimens should be considered as a potential factor contributing to the observed high CSTAdV detection rate. Several other Chinese soft-shelled turtle-associated viral pathogens were also tested in these clinical samples from diseased Chinese soft-shelled turtles, including STIV and TSHSV. No STIV was detected, and only two TSHSV cases were identified. The severity of adenovirus infections ranges from subclinical to lethal, and the clinical symptoms of adenovirus-infected animals showed diversity [1, 2]. This suggests that adenovirus may be an important contributing factor to the varied clinical symptoms observed in the diseased turtles.

## Conclusions

This study is the first to establish CSTAdV RF-RAA and qPCR assays for the epidemiological investigation and disease prevention and control of turtle adenovirus infections. The RF-RAA assay is suitable for pathogen screening in the field, while the qPCR assay is ideal for quantitative analysis in laboratory settings. Both assays are very effective for detecting CSTAdV, and testers can select the appropriate method based on specific requirements or combine the two methods.

## Data Availability

No datasets were generated or analysed during the current study.
